# Design and Implementation of an Inpatient Fall Risk Management Information System

**DOI:** 10.2196/46501

**Published:** 2024-01-02

**Authors:** Ying Wang, Mengyao Jiang, Mei He, Meijie Du

**Affiliations:** 1 School of Management Wuhan University of Technology Wuhan China; 2 Department of Nursing Tongji Hospital Tongji Medical College, Huazhong University of Science and Technology Wuhan China

**Keywords:** fall, hospital information system, patient safety, quality improvement, management, implementation

## Abstract

**Background:**

Falls had been identified as one of the nursing-sensitive indicators for nursing care in hospitals. With technological progress, health information systems make it possible for health care professionals to manage patient care better. However, there is a dearth of research on health information systems used to manage inpatient falls.

**Objective:**

This study aimed to design and implement a novel hospital-based fall risk management information system (FRMIS) to prevent inpatient falls and improve nursing quality.

**Methods:**

This implementation was conducted at a large academic medical center in central China. We established a nurse-led multidisciplinary fall prevention team in January 2016. The hospital’s fall risk management problems were summarized by interviewing fall-related stakeholders, observing fall prevention workflow and post–fall care process, and investigating patients' satisfaction. The FRMIS was developed using an iterative design process, involving collaboration among health care professionals, software developers, and system architects. We used process indicators and outcome indicators to evaluate the implementation effect.

**Results:**

The FRMIS includes a fall risk assessment platform, a fall risk warning platform, a fall preventive strategies platform, fall incident reporting, and a tracking improvement platform. Since the implementation of the FRMIS, the inpatient fall rate was significantly lower than that before implementation (*P*<.05). In addition, the percentage of major fall-related injuries was significantly lower than that before implementation. The implementation rate of fall-related process indicators and the reporting rate of high risk of falls were significantly different before and after system implementation (*P*<.05).

**Conclusions:**

The FRMIS provides support to nursing staff in preventing falls among hospitalized patients while facilitating process control for nursing managers.

## Introduction

### Context

Falls are one of the nursing-sensitive indicators for nursing care [1], which are a leading cause of fatal and nonfatal health loss globally [2,3]. Reducing and preventing falls has become an international health priority. Falls—common adverse events reported in hospitals—have been identified as a nursing-sensitive quality indicator of patient care.

Given the growing technological progress, health IT may help enhance the quality and safety of provided care, facilitating the effectiveness and efficiency of the clinical workflow, and supporting the provision of integrated multidisciplinary care [4-11]. The hospital information system (HIS) is a promising approach to improve care quality and safety in the complex hospital environment. Despite extensive literature on fall risk factors and preventive strategies [12-18], few studies have focused on health information systems for managing inpatient falls.

### Problem Statement

To address these issues, we formed a nurse-led multidisciplinary fall prevention team in January 2016, including the hospital administrative staff, quality management specialists, physicians, nurses, pharmacists, and informatics staff. This team retrospectively analyzed 19 inpatient fall cases that occurred in 2015 (fall rate 0.015‰), ranking first among all in-hospital nursing adverse events. Among the fall cases, 30%-40% of patients had grade ≥3 injuries, which significantly exceeded the 3.978% proportion seen in similar hospitals during this period. Falls caused severe harm and financial burden to inpatients, with 3 patients experiencing severe head injuries and 2 having hip fractures. The longest hospital stay resulting from falls reached 36 days.

The hospital’s fall risk management problems were summarized by interviewing fall-related stakeholders, observing fall prevention workflow and postfall care process, and investigating patients' satisfaction; these included (1) nonachievement of real-time fall risk assessment, real-time uploading, and information sharing; (2) absence of fall risk warning management; (3) complicated fall risk management workflow; (4) absence of process control in fall prevention (such as process control for different fall risk levels, process control for different time nodes, etc); and (5) lack of standardized pathways for inpatient fall incident reporting and improvement tracking.

### Similar Interventions

Several studies have highlighted the benefits of using health information systems for patient fall management. For example, Giles et al [19] reported that data collected from nursing information systems can be used to identify high-fall-risk patients. Mei et al [20] designed an electronic patient fall reporting system in a US long-term residential care facility, which could improve the fall reporting process and subsequent quality improvement efforts. Katsulis et al [21] combined the Fall TIPS (Tailoring Interventions for Patient Safety) [22] with a clinical decision support system, which increased its ease of use over the paper version. Jacobsohn et al [23] developed an automated clinical decision support system for identifying and referring older adult emergency department patients at the risk of future falls. Mlaver et al [24] at the Brigham and Women’s hospital developed a valuable electronic health record–embedded dashboard that collected inpatient fall risk data. However, the abovementioned fall information system only focused on a specific domain of fall management. So far, there is still no report about an HIS for overall fall risk management.

### Aims and Objectives

This implementation aims to design and implement a fall risk management information system (FRMIS) to reduce falls among inpatients and improve nursing quality. Our goal is to create a culture of safety and reduce the incidence of falls hospital-wide, ensuring the well-being and security of all patients.

## Methods

This study adhered to the iCHECK-DH (Guidelines and Checklist for the Reporting on Digital Health Implementations) checklist [25].

### Blueprint Summary

This FRMIS consists of 4 major functional platforms to facilitate comprehensive fall prevention pathway management as shown in Textbox 1.

**The 4 major functional platforms of the fall risk management information system.**

**A fall risk assessment platform (Multimedia Appendix 1):**

The assigned nurse uses a personal digital assistant to conduct fall risk assessments within 4 hours of patient admission. Upon completion, the personal digital assistant automatically compiles the Morse Fall Risk Score [26,27] and risk level, marking it in the electronic nursing record. All patients' Morse Fall Risk Scores are collected and shared in real time through the information platform. Simultaneously, nurses receive nursing guidelines specific to different fall risk levels. They implement corresponding fall prevention measures such as hanging “Fall Prevention” warning signs near high-risk patients' beds, distributing “Fall Prevention Information Sheets” to guide patients and their families on preventive measures, and documenting and passing on relevant information during shift changes. The head nurse conducts daily inspections and guidance on the accuracy of Morse fall assessments and the implementation of fall prevention measures, upon completion of departmental reviews.

**A fall risk warning platform (Multimedia Appendix 1):**

Patients at different fall risk levels are color-coded for easy identification: high-risk (Morse Fall Risk Score≥45), red; moderate (score approximately 25-44), yellow; and low (score approximately 0-24), green. The fall risk warning module comprehensively displays the daily number of high-risk falls, department distribution and ranking, percentage of the population at the risk of falls, specific bed locations, medical diagnoses, Morse Fall Risk Scores, and assessment times through charts and color-coded indicators. This provides nursing managers real-time insights into the key populations, departments, and information related to fall risk management, enabling proactive fall risk prevention and providing precise information support for effective fall prevention process control.

**A fall preventive strategies platform (see Multimedia Appendix 1):**

Evidence-based fall prevention strategies are developed, incorporating fall event analysis and expert discussions to extract key process monitoring indicators. An electronic fall prevention bundle strategies quality tracking checklist was established for accurate assessment of fall risk, increased awareness of preventive measures, enhanced handover process for high-risk patients, environmental safety, implementation of fall prevention knowledge training, and guidance on proper use of assistive devices. Nursing department and ward-level managers can use mobile devices (iPads) to conduct targeted goal management and quality inspections of fall prevention strategies. Real-time monitoring is conducted on key fall process indicators such as accuracy of Morse fall risk assessments, implementation of health education, adherence to handover procedures, and compliance with environmental safety measures.

**A fall incident reporting and tracking platform (see Multimedia Appendix 1):**

The platform regulates the reporting process for inpatient fall events. After a fall incident occurs, the ward head nurse promptly logs into the fall incident reporting platform to proactively report the incident. They provide details such as the time and location of the fall, the sequence of events, whether the patient was injured, the extent of the injury, and the emergency treatment process. Once the information platform receives the ward's report, it immediately sends text messages to the chief nurse and members of the nursing department's safety management team. On the platform, safety management team members can quickly trace the Morse Fall Risk Score, risk level, appropriateness of fall prevention interventions, timeliness of assessments, and any dynamic evaluations associated with that patient. After gaining a comprehensive understanding of the patient's relevant information, they visit the ward in a timely manner to conduct on-site inspections and tracking. They provide guidance to the department by applying root cause analysis to thoroughly analyze the fall event, identify the underlying causes, and propose areas of improvement directly on the web-based platform. Ward head nurses and the chief nurse can access expert guidance instantly on the platform and make necessary improvements based on the advice provided.

### Technical Design

The FRMIS was developed using an iterative design process, involving collaboration among health care professionals, software developers, and system architects. The design aimed to create a user-friendly interface, incorporate data integration capabilities, and enable real-time reporting functionalities. In order to meet the usage needs of both PC and mobile devices, the development language selected for this system includes C#, jQuery, and Java; the development tools used were Visual Studio (Microsoft Corp) and Eclipse (The Eclipse Foundation), and the development platforms used were Windows and Android.

### Target

The FRMIS was designed to assist nursing staff in preventing inpatient falls through IT, facilitating process control for nursing managers and ensuring patient safety.

### Data

Our hospital has a dedicated computer center, which serves as the technical support department for network security. It is responsible for the construction and operation of hospital network security protection measures. The collection of various data in the FRMIS complies with relevant national laws and regulations. The data collection scope follows the principle of “minimum necessary” and adopts measures such as data desensitization, data encryption, and link encryption to prevent data leakage during the data collection process.

### Interoperability

To maximize the effectiveness of the FRMIS, standardization of data elements and the development of interface systems to allow seamless data exchange between our HISs were necessary. The FRMIS used Health Level Seven Fast Healthcare Interoperability Resources (HL7FHIR) to enable seamless data exchange and streamline workflows.

### Participating Entities

The FRMIS project has obtained the approval and support of hospital management, who have provided strong guarantees in terms of personnel, resources, funding, and working hours required for the implementation of the research plan. Our hospital is an advanced information management hospital with state-of-the-art scientific technologies. The computer center has rich experience in developing information management platforms; they have independently developed and implemented 19 hospital operational management systems. The FRMIS’s development was initiated by the nursing department, with the assistance of the computer center to fulfill the corresponding requirements.

### Budget Planning

The FRMIS development process lasted about 4 months, and the total development cost was approximately 500,000 Renminbi (approximately US $68,300). The subsequent maintenance costs were estimated to be 8% of the total development cost annually. Funding for the FRMIS’s development and maintenance was provided by our hospital. The ownership of the FRMIS belongs to Tongji Hospital.

### Sustainability

The FRMIS’s implementation was carried out through the issuance of relevant policy documents by the nursing department, ensuring its clinical adoption. All risk assessment and incident reports concerning the inpatient falls were conducted through this information system thus far, replacing the previous paper-based forms. Over the past few years of using this system, our hospital's computer center staff has been maintaining and fixing occasional bugs that occur during clinical implementation of this system. The computer center staff also made necessary modifications and improvements to certain details as needed to enhance system functionality, optimize workflows, and adapt to evolving health care practices.

### Statistical Analysis

Statistical comparisons were made on the fall incidence rate among inpatients and the reporting rate of high-fall-risk patients before and after FRMIS implementation. Data entry and statistical analysis were performed using SPSS (version 17.0; IBM Corp). The chi-square test was used to compare the differences in the fall incidence rate among inpatients, the rate of high-fall-risk patients, and the implementation rate of preventive fall quality bundle strategy indicators before and after FRMIS implementation. A value of *P*<.05 was considered statistically significant.

### Ethics Approval

The study was approved by the institutional review board of Tongji hospital (protocol TJ-IRB20191209).

## Implementation (Results)

### Coverage

Our hospital is a large academic medical center in central China. In 2016, the hospital had a total of 4000 open beds, 106 nursing wards, and 53 specialized nursing units. The average daily admission rate ranges from 4500 to 5000 patients, with a total of 193,709 admitted patients throughout the year. The cumulative number of bed-days reached 1,756,946, of which 277,365 (15.79%) were for critical patients.

### Outcomes

We carried out the process and outcome evaluation with regard to the FRMIS’s implementation. The process evaluation indicators include (1) the accuracy rate of the Morse fall risk assessment: number of accurate Morse fall risk assessments / total number of Morse fall risk assessments inspected; (2) implementation rate of fall prevention health education: number of implemented health education check items / number of patients inspected × total number of fall prevention health education check items; (3) implementation rate of shift handoff: number of implemented shift handoff check items / number of patients inspected × total number of fall prevention shift handoff check items; (4) implementation rate of environment safety: number of implemented environment safety check items / the number of patients inspected × the total number of environment safety check items.

The staff of the quality control office in the nursing department reviewed the FRMIS on a daily basis to identify the clinical departments where high-fall-risk patients were distributed across the hospital. For departments with more than 5 high-fall-risk patients and a proportion exceeding 20% of the total patients, we assigned 2 supervisory staff from the quality control team. They used the electronic form “Fall Prevention Bundle Strategy Quality Tracking Form” (see Multimedia Appendix 1) on an iPad to conduct quality inspections on the nursing units for the high-fall-risk patient population, randomly checking the implementation rate of fall prevention bundle strategy indicators (fall risk assessment, fall-related health education, fall-related shift handoff, and environment safety). Before implementing the FRMIS, a total of 1250 patients were randomly sampled for inspection. After implementing FRMIS, a total of 1806 patients were randomly sampled for inspection. Additionally, a comparative analysis was performed on the hospitalization period between February and October 2017 (after FRMIS implementation, the total bed days occupied by inpatients was 1,323,667) and between February and October 2015 (before FRMIS implementation, the total bed days occupied by inpatients was 1,303,094) to evaluate the hospital-wide reporting rate of high-fall-risk cases, incidence rate of patient falls, and severity of fall-related injuries.

The results showed that since the FRMIS’s implementation, the inpatient falls rate was significantly lower than that before implementation (*P*<.001), as shown in Table 1. In addition, the percentage of major fall-related injuries was significantly lower than that before implementation, as shown in Table 2. The implementation rate of fall-related process indicators and the reporting rate of high risk of falls were significantly different before and after system implementation (*P*<.001), as shown in Table 3.

**Table 1:** Comparison of fall-related outcome indicators.

	Before implementation (total bed days=1,303,094), n (%)	After implementation (total bed days=1,323,667), n (%)	Chi-square (*df*)	*P* value
High-fall-risk patients’ reports	1036 (0.8)	3007 (2.3)	931.7 (1)	<.001
Fall incident reports	23 (0.02)	11 (0.01)	4.4 (1)	<.001

**Table 2:** Results of fall-related injuries.

	Cases of fall-related injury, n			
	No injury	Minor	Moderate	Major
Before implementation	15	28	12	2
After implementation	20	13	9	0

**Table 3:** Comparison of fall-related process indicators.

	Before implementation (n=1250), n (%)	After implementation (n=1806), n (%)	Chi-square (*df*)	*P* value
Fall risk assessment	1056 (84.48)	1709 (95.73)	88 (1)	<.001
Fall-related health education	1107 (88.56)	1769 (97.95)	117.5 (1)	<.001
Fall-related shift handoff	1114 (89.12)	1767 (97.84)	104 (1)	<.001
Environment safety	1127 (90.16)	1796 (99.45)	153 (1)	<.001

### Lessons Learned

The FRMIS’s development and implementation followed a structured process, starting with needs assessment and culminating in ongoing monitoring and improvement. With this multidisciplinary team and comprehensive approach, we were able to provide a more robust and effective fall risk management system for the entire hospital. The FRMIS addressed the shortcomings of paper-based reporting, such as untimely fall assessments, delayed reporting, information transmission delays, loss of assessment forms, and incomplete tracking information. The FRMIS achieved a holistic fall prevention strategy that spanned from risk assessment to postfall intervention, which brought several benefits to both patients and health care providers. The FRMIS alerted nursing staff about high-risk patients, enabling timely interventions and reducing fall occurrences. It also standardized the reporting process for fall events, allowing for efficient tracking and analysis of incidents.

## Discussion

### Principal Findings

This study has designed and implemented an FMRIS at the hospital level. The novel system provided a simple, intuitive, and highly operational prevention management model, encompassing fall risk assessment, high fall risk screening, forecasting, and monitoring. It significantly improved the procedural and standardized levels of fall management for hospitalized patients, having prompted nurses to proactively implement fall preventive interventions, conducted timely fall risk assessments, reduced underreporting of high-fall-risk patients, and increased the forecast rate of high-fall-risk patients.

Unlike previous studies that focus on a specific stage of fall management (such as risk identification [19] or fall incident reporting [28]) or patients in a specific department [23], our system catered to the entire process of fall risk management for all inpatients. The FRMIS showed promise in enhancing patient safety, reducing fall incidents, and improving overall care quality.

To facilitate the successful implementation of the FRMIS in clinical practice, we first developed the Standardized Management Guidelines for Preventing Inpatient Falls at the hospital-wide level. This policy document comprehensively revises and improves clinical fall prevention efforts, which include patient fall risk assessment, health education, fall preventive interventions, fall management workflow, fall incident reporting, and system record-keeping. The policy document was distributed in hard copy by the nursing department to all departments and also uploaded electronically on the hospital's Office Automation platform. It mandated each clinical department to conduct fall prevention training based on the guidelines, requiring all nurses’ participation and proficiency. This document served as a supporting tool, providing nurses with guidance on how to use the FRMIS effectively in their clinical practice to prevent inpatient falls.

In addition, we conducted standardized nurse training through a web-based platform. Three main implementation strategies were used. First, we conducted diverse forms of training, including ward-, department-, and hospital-level fall prevention training, as well as case-based warning education, bedside simulation assessment, experience sharing sessions, and special lectures, to comprehensively implement the content of the Standardized Management Guidelines for Preventing Falls. Second, we performed objective evaluation. We incorporated simulated case examinations for patient fall prevention into the clinical skills evaluation of nurses, head nurse position evaluation, and their performance appraisal to comprehensively assess the level of knowledge of fall management guidelines and the emergency handling capabilities for patient fall incidents. Third, we achieved full participation among all nurses. The training rate and assessment results of nurses in the wards were included in the performance management projects of ward head nurses, achieving the participation of all nurses and comprehensive evaluation of standardized fall prevention training. Based on the strategies mentioned above, the FRMIS’s implementation in clinical practice has been relatively successful.

### Limitations

This study still has certain limitations that should be acknowledged. First, the FRMIS was specifically designed and implemented by our hospital's computer center. It is currently applicable to 3 different hospital campuses within our institution but has not been widely disseminated to other hospitals or integrated with diverse HISs. Therefore, its applicability and effectiveness in different hospital contexts remains uncertain. Second, the FRMIS heavily relied on the voluntary reporting by clinical nurses. The accuracy of these fall risk reports needed to be individually verified by staff members in the quality control office of the nursing department. This process is currently manual and lacks automation, which may introduce delays and potential inconsistencies. In the future, further improvements could be made by integrating artificial intelligence (AI) technologies. By automatically extracting fall risk factors from patients' electronic medical records, the system could achieve automated risk stratification and reduce dependence on manual reporting.

Despite these limitations, it is important to note that this study represents a significant step toward enhancing inpatient fall risk management through the FRMIS implementation. Future research and development efforts could focus on expanding the system's applicability to other hospitals, integrating AI capabilities for automated risk assessment, and improving data accuracy and automation processes. These advancements would contribute to more comprehensive and intelligent fall risk management practices for inpatients.

### Conclusions

The design and implementation of an FRMIS significantly contributed to the prevention and management of falls among inpatients. The FRMIS enhanced patient safety through IT, providing comprehensive support for fall prevention and ensuring efficient management of fall events in health care settings.

## Appendix

Multimedia Appendix 1 The presentation of the fall risk management information system.

## Abbreviations

AI: artificial intelligence
FRMIS: fall risk management information system
HIS: hospital information system
HL7FHIR: Health Level Seven Fast Healthcare Interoperability Resources
iCHECK-DH: Guidelines and Checklist for the Reporting on Digital Health Implementations
TIPS: Tailoring Interventions for Patient Safety

## Data Availability

The data sets used or analyzed during this study available from the corresponding author on reasonable request.
