# Supplementary material for: Design and Implementation of an Inpatient Fall Risk Management Information System
Source: JMIR Med Inform. 2024 Jan 2;12:e46501. doi: 10.2196/46501 (PMC10792483; doi:10.2196/46501)
Supplement: Multimedia Appendix 1 [file medinform_v12i1e46501_app1.docx]

**Appendix**

**
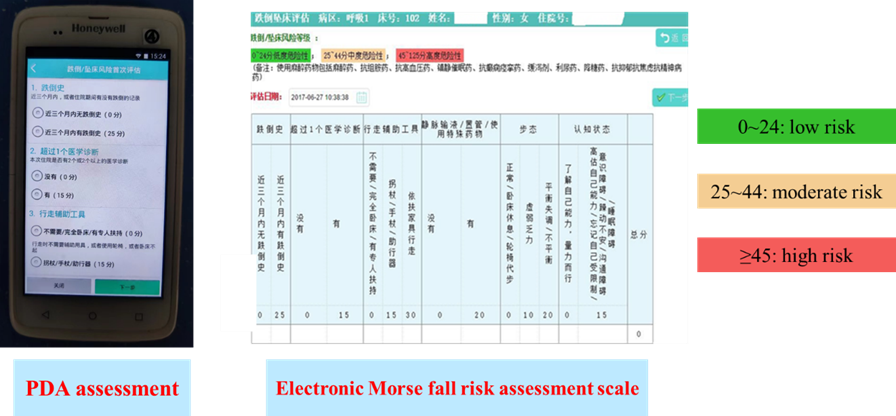
**

Screenshot of the fall risk assessment platform


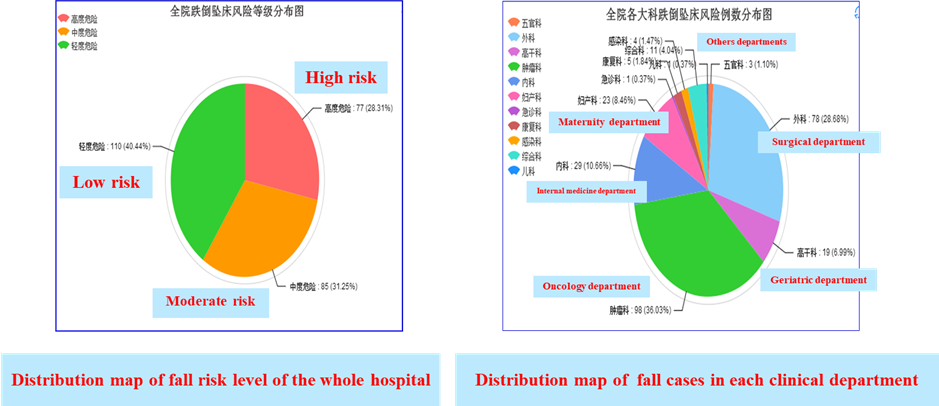


Fall risk warning platform content


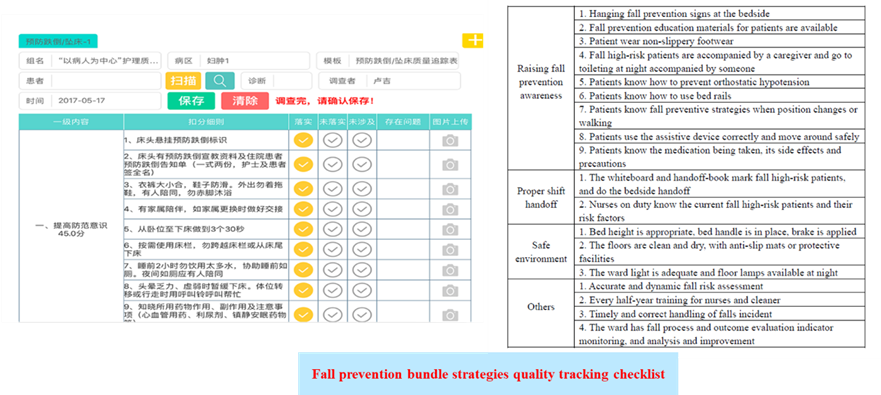


Screenshot of the fall preventive strategies platform


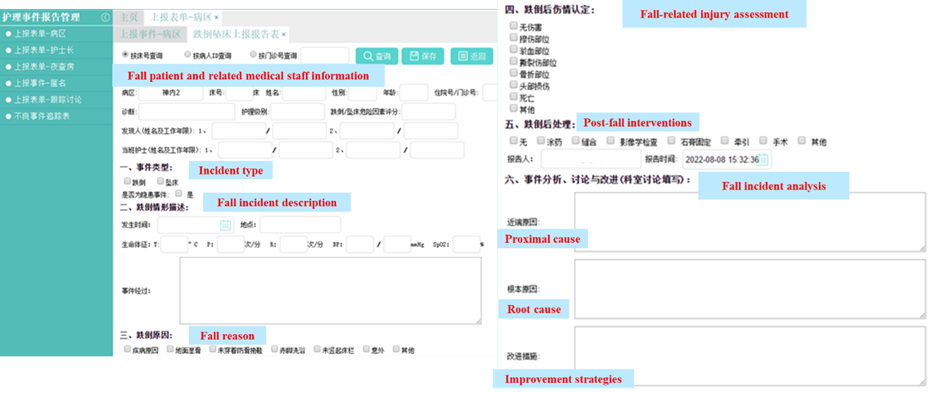


Screenshot of the fall incident reporting platform

**Fall prevention bundle strategies quality tracking checklist**

| Raising fall prevention awareness | 1. Hanging fall prevention signs at the bedside |
| --- | --- |
|  | 2. Fall prevention education materials for patients are available |
|  | 3. Patient wear non-slippery footwear |
|  | 4. Fall high-risk patients are accompanied by a caregiver and go to toileting at night accompanied by someone |
|  | 5. Patients know how to prevent orthostatic hypotension |
|  | 6. Patients know how to use bed rails |
|  | 7. Patients know fall preventive strategies when position changes or walking |
|  | 8. Patients use the assistive device correctly and move around safely |
|  | 9. Patients know the medication being taken, its side effects and precautions |
| Proper shift handoff | 1. The whiteboard and handoff-book mark fall high-risk patients, and do the bedside handoff |
|  | 2. Nurses on duty know the current fall high-risk patients and their risk factors |
| Safe environment | 1. Bed height is appropriate, bed handle is in place, brake is applied |
|  | 2. The floors are clean and dry, with anti-slip mats or protective facilities |
|  | 3. The ward light is adequate and floor lamps available at night |
| Others | 1. Accurate and dynamic fall risk assessment |
|  | 2. Every half-year training for nurses and cleaner |
|  | 3. Timely and correct handling of falls incident |
|  | 4. The ward has fall process and outcome evaluation indicator monitoring, and analysis and improvement |
